# Deformation Properties of Rubberized ECC Incorporating Nano Graphene Using Response Surface Methodology

**DOI:** 10.3390/ma13122831

**Published:** 2020-06-24

**Authors:** Dexter Ling Hau Hong, Bashar S. Mohammed, Amin Al-Fakih, Mubarak Mohammed A Wahab, M. S. Liew, Y. H. Mugahed Amran

**Affiliations:** 1 Civil and Environmental Engineering Department, Universiti Teknologi PETRONAS, Bandar Seri Iskandar 32610, Malaysia; uitm1314@gmail.com (D.L.H.H.); amin.ali_g03663@utp.edu.my (A.A.-F.); mubarakwahab@utp.edu.my (M.M.A.W.); shahir_liew@utp.edu.my (M.S.L.); 2 Department of Civil Engineering, College of Engineering, Prince Sattam Bin Abdulaziz University, Alkharj 11942, Saudi Arabia; m.amran@psau.edu.sa; 3 Department of Civil Engineering, Faculty of Engineering and IT, Amran University, Quhal, Amran 9677, Yemen

**Keywords:** crumb rubber (CR), graphene oxide (GO), response surface methodology (RSM), drying shrinkage, engineered cementitious composite (ECC)

## Abstract

Engineered cementitious composite (ECC) was discovered as a new substitute of conventional concrete as it provides better results in terms of tensile strain, reaching beyond 3%. From then, more studies were done to partially replace crumb rubber with sand to achieve a more sustainable and eco-friendlier composite from the original ECC. However, the elastic modulus of ECC was noticeably degraded. This could bring potential unseen dangerous consequences as the fatigue might happen at any time without any sign. The replacement of crumb rubber was then found to not only bring a more sustainable and eco-friendlier result but also increase the ductility and the durability of the composite, with lighter specific gravity compared to conventional concrete. This study investigated the effects of crumb rubber (CR) and graphene oxide (GO) toward the deformable properties of rubberized ECC, including the compressive strength, elastic modulus, Poisson’s ratio, and drying shrinkage. Central composite design (CCD) was utilized to provide 13 reasonable trial mixtures with the ranging level of CR replacement from 0–30% and that of GO from 0.01–0.08%. The results show that GO increased the strength of the developed GO-RECC. It was also found that the addition of CR and GO to ECC brought a notable improvement in mechanical and deformable properties. The predicted model that was developed using response surface methodology (RSM) shows that the variables (compression strength, elastic modulus, Poisson’s ratio, and drying shrinkage) rely on the independent (CR and GO) variables and are highly correlated.

## 1. Introduction

Engineered cementitious composite (ECC) is classified as a high-performance fiber-reinforced cementitious composite (HPFRCC). It is known for its outstanding pseudo-strain-hardening behavior and ultra-high ductility. This is due to its proper interfacial bond between fibers and the surrounding cementitious matrix [1]. ECC was developed into many forms including self-consolidating ECC [2], high-early-strength ECC, sprayable ECC, lightweight ECC, green ECC, and self-sensing and self-healing ECC. All these forms were designed differently in terms of their energy dispersion (designed for seismic impact and blast resistance) and high fatigue resistance (for bridges, railways, and roads) [3].

The advantages of ECC were found to be its ultra-high toughness, multiple micro-cracking behaviors, better fatigue resistance, good durability, and self-healing characterization [4]. According to Şahmaran and Li [5], the addition of fly ash (FA) is also one of the essential components in ECC as the increment of FA in ECC reduces the crack width from about 60 μm to 10–30 μm, contributing positively toward the durability of the structure in long-term periods. ECC only shows high tensile ductility with a moderate fiber volume fraction (normally 2%) is added to ECC [6,7].

However, it was found that ECC shows signs of ultra-high toughness and good durability, but it shows degradation of the elastic modulus. ECC provides an increment in tensile strain capacity by around 3–8% [7]. However, the high tensile strain in ECC also brings higher possibility of sudden failure as the damage tolerance is reduced as the tensile strain increases [8]. Moreover, ECC also has its drawbacks including lower compressive strength (40.8 MPa) compared to conventional concrete (49.7 MPa). It was also shown that, following the addition of high-volume fly ash (HVFA), there is an increase in fire resistance, fiber/matrix chemical bond interface, matrix toughness, drying shrinkage, tensile strain capacity, multiple cracking, and crack width, but it attains a decrease in compressive, flexural, and tensile strength [8]. 

Crumb rubber is proven to have lower strength, water absorption, and stiffness [9,10]. According to Siad et al. [11], it was found that coarse rubber sand significantly increases the deflection capacity of ECC mixtures at the optimum content of 20%. The authors also reported that the addition of crumb rubber into ECC increased the drying shrinkage of ECC. 

However, the use of crumb rubber unfavorably affects the compressive strength and flexural strength of ECC. It was investigated that replacing only 10% of fine sands with crumb rubber brought a great degradation of up to 63% in compressive strength [12]. The reason for the reduction in strength is because of the hydrophobic properties of crumb rubber, enabling air entrapment on the surface of the crumb rubber and repelling water during the mixing process [13,14]. 

According to Huang et al. [12], the modulus of elasticity decreases as the percentage of crumb rubber replacement in ECC increases. The study found that elastic modulus was reduced by 50% following replacement with 10% fine sand. Essential factors that contribute to the degradation of the elastic modulus in rubberized ECC include higher void content in the cement paste, weak bonding between cement paste and crumb rubber particles, and thicker and weaker interfacial transition zone (ITZ) due to air entrapped by crumb rubber, which significantly influences the stress–strain behavior [15]. A study by Zhang et al. [16] discovered that the drying shrinkage of ECC containing crumb rubber particles increased from 1050 × 10^−6^ to 1660 × 10^−6^ at the 90th day. In addition, the lower strength and elastic modulus and the higher water to cement ratio contribute to the lower susceptibility to drying of rubberized ECC [15]. Despite the result of high drying shrinkage being identified in rubberized ECC, it still has relatively low drying shrinkage compared to unmodified ECC (1200 × 10^−6^ to 1800 × 10^−6^), causing the rubberized ECC to be able to withstand shrinkage-induced deformation without initiating localized fracture [16].

Graphene oxide (GO) is a hydrophilic material, having the capacity to form stable H-bonds with silicate hydroxyl and calcium hydroxyl groups near the surface of cementitious material [11]. Pan et al. [17] found that incorporating only 0.03% GO sheets into cementitious composite can actually dramatically increase its compressive strength and tensile strength by up to 40% due to the reduction of pores in the cementitious composite. By adding GO to ECC, results showed an increment of tensile strength of 197.2% and an increment of compressive strength of 160.1% with 0.02 wt.% GO [18]. Furthermore, a 500% increase in elastic modulus with 3% GO was recorded [19]. Mohammed et al. [20] concluded that GO has the potential to refine the microstructure of cementitious materials by increasing the number of gel pores and decreasing the number of capillary pores, thereby altogether efficiently enhancing the mechanical strength of cement composite. Furthermore, according to Sharma et al. [21] and Sharma and Kothiyal [22], it was concluded that the total porosity of cementitious composites with 1% GO can be reduced from 25.21% to 10.61%. 

Despite all the advantages brought by ECC, additional studies were also done to further improve the ECC properties. It was found that the elastic modulus of unmodified ECC is low, resulting in the ECC structure having a low resistance toward elastic deformation. However, despite the concept of integrating crumb rubber particles into cement-based material being applied for decades, it still has disadvantages such as lower compressive and tensile strength. In order to overcome the drawbacks of crumb rubber in ECC, graphene-filled cementitious composite was found to be the ideal nano-filler to modify the cementitious material composite as it provides strong bonding to oxygen functional groups. Therefore, this study aims to investigate the mechanical and deformation properties of modified ECC incorporating CR and GO. Moreover, statistical analysis was carried out using response surface methodology (RSM) to validate the experimental results and, therefore, develop a model for easier design that can predict the properties of ECC mixtures.

## 2. Material Properties and Methodological Program

**Figure 1:**
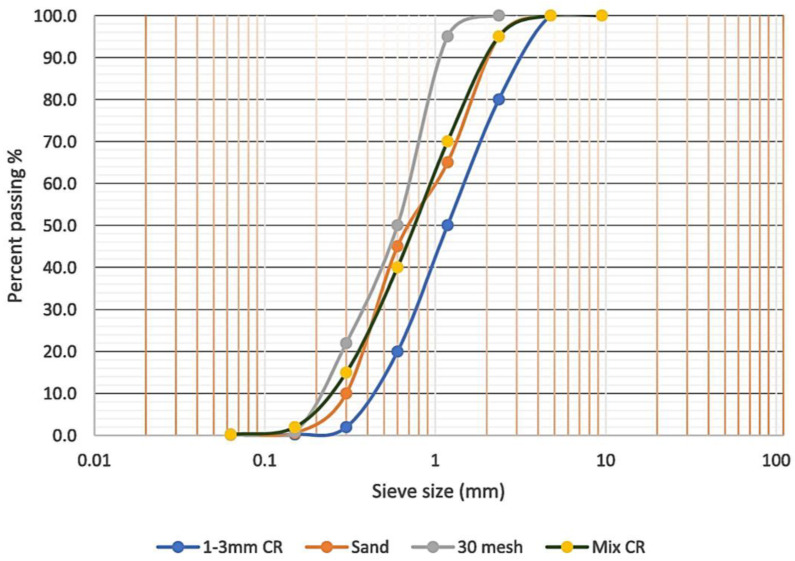
Grading curve of fine sand and crumb rubber.

The materials utilized in the development of graphene oxide-modified rubberized ECC (GO-RECC) were sand, crumb rubber, fly ash, ordinary Portland cement (OPC), polyvinyl alcohol (PVA) fibers, and water. OPC was of Type I that confirmed the requirements of ASTM C150 [23]. Class F fly ash (FA) was in accordance with the requirements specified in ASTM C618-17 [24] with a density of 1300 kg/m^3^, an amount of Al_2_O_3_ + Fe_2_O_3_ + SiO_2_ of 82.12%, and below 6% loss on ignition. FA was utilized in GO-RECC to reduce the cost of the material, as it behaves as an intense water-reducing substance. FA is a by-product of pulverized coal being burned in thermal electric generation plants, and it is a waste material which has pozzolanic properties, resulting in it being classified as a cement-replacing material. The chemical contents of OPC and FA are presented in Table 1. Polyvinyl alcohol (PVA) fibers were added to the mixtures with the volume fixed at 2% to achieve uniform dispersion and workability, as well as to adhere to the principles of micromechanics requirements, in order to improve ductility and impart high strain in a cementitious matrix. The details of the PVA fibers are shown in Table 2. Local washed river sand was used in the mixes conforming to ASTM C33-M16 [25]. The sizes of 0.3–1.18 mm and a sand/cementitious ratio of 0.36 were utilized to maintain enough stiffness and volume stability to obtain better fresh and hardened properties of GO-RECC. CR particles were varied from 0–10% and used as a partial replacement of sand by volume with a combination size of sieve 30 mesh and sieve size of 1 to 3 mm in the appropriate mixed proportions of 60% and 40% [26]. In order to attain a similar trend as that of sand particles where the sand is replaced with the crumb rubber, the final gradation of crumb rubber contained 60% of passing size #30 mesh and 40% of size passing 1–3 mm. The specific gravity of crumb rubber is 0.95, which replaced the amount of fine aggregate by volume percentage. The sieve analysis of the fine aggregate and crumb rubber is shown in Figure 1. GO with a concentration of 4 mg/mL was utilized, and the final composition ranged from 0.01–0.05% by volume. The physical properties of GO and its elemental analysis are shown in Table 3 and Table 4. An aqueous solution of superplasticizer known as modified polycarboxylate-based (HRWR) “Sika Viscocrete-2044” was used to adjust the mixtures to accomplish the desired flowability. Sika Viscocrete-2044 is a polycarboxylate superplasticizer (SP) in liquid form with a pH of 6.2 and 1.08 specific gravity, with an absence of chloride ion content. Water that is suitable for drinking is usually considered acceptable for mixing concrete. In this study, the water-to-cement ratio was set to 0.215.

Through response surface methodology (RSM), a graphical response was provided for visually determining the independent variables (CR and GO) influencing the responses. An approximate solution of the responses (compressive strength, elastic modulus, Poisson’s ratio, drying shrinkage) was obtained, and the optimization of the response surface was conducted for the best solution. RSM was adopted to provide 13 ECC mixes, and then the developed mixtures were tested at 28 days for hardened properties including compressive strength, drying shrinkage, elastic modulus, and Poisson’s ratio. Consequently, optimized mixture proportions for the graphene oxide-modified rubberized ECC (GO-RECC) were determined. The 13 mixtures with three different proportions of GO (0.01%, 0.03%, and 0.05%) by volume and three levels of crumb rubber replacement (0%, 5%, and 10%) to fine aggregate were considered, as shown in Table 5.

**Figure 2:**
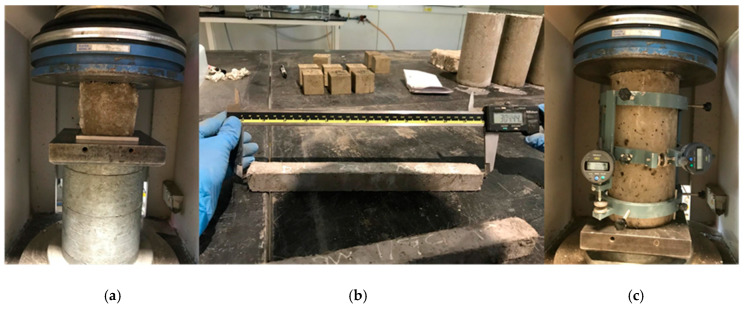
Hardened samples and testing set-up. (**a**) Compressive strength test, (**b**) Drying shrinkage test, and (**c**) Elastic modulus and Poisson’s ratio test.

The compressive strength test was conducted by using the 13 trial mixes of the GO-RECC mixture cubes with dimensions of 50 mm × 50 mm × 50 mm (Figure 2a). Three samples per mix were tested according to BS 1881: Part 116:1983 [27]. For the drying shrinkage test, three prisms per mix with the dimensions of 75 mm × 75 mm × 300 mm (Figure 2b) were used in accordance with ASTM C596-01 [28]. Shrinkage deformation was defined as a change in length of the specimens from the beginning of the experiment to the air-drying age of 28 days. To obtain the elastic modulus and Poisson’s ratio of the mixtures, cylindrical molds with 150 mm diameter and 300 mm length (Figure 2c) were tested as per the requirement of ASTM C469-14 [29].

## 3. Results and Discussion

### 3.1. Responses Results

In this study, Design-Expert software version 11 (Stat-Ease, Inc., Minneapolis, MN, USA) was implemented, and the deformation properties and compressive strength obtained through experiments were used to develop response prediction models and perform the least square regression analysis. The design matrix with manipulated variables in this study is displayed in Table 6, along with experimental result values.

### 3.2. Compressive Strength of GO-RECC

**Figure 3:**
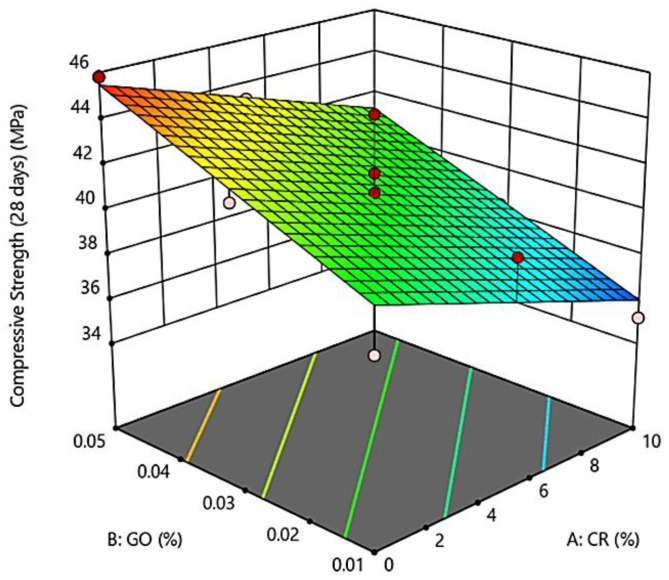
Three-dimensional (3D) surface response for the compressive strength of GO-RECC.

Figure 3 illustrates the three-dimensional (3D) surface response analysis of the developed GO-RECC. These indicate the deformable effects of CR, as a partial replacement of fine aggregates, and GO, as a filler material, on the compressive strength of GO-RECC.

As shown in Figure 3, the average compressive strength of the modified ECC was higher without the addition of CR. According to the experimental results, the compressive strength of GO-RECC decreased by about 3.5% with just the addition of CR with 5% compared to the mix with 0% CR. This reduction in strength was due to the increment of porosity through the hydrophobic nature of CR, repelling water and causing air entrapment in the ECC microstructure [12]. Moreover, it is known that CR has an original elastic modulus with a range from 1.3 MPa to 5.3 MPa, which is lower compared to the cement paste. This is the main factor that caused a noticeable decrease in strength due to the non-polar nature and mechanical flexibility of the CR. 

On another hand, although the strength of GO-RECC reduced, it was still higher than that of normal rubberized ECC due to the addition of PVA fiber with a fixed 2% of the weight of cementitious material. The randomly distributed fibers strengthened the ECC and controlled cracking propagation, thereby increasing the compressive strength [30]. Furthermore, the addition of GO also resulted in a noticeable increment in compressive strength. It is believed that the presence of GO forms flower-like shaped hydration crystals, and this causes the dispersion of all cement particles in void spaces, resulting in the enhancement of not only flexural but also compressive strength [31].

### 3.3. Modulus of Elasticity of GO-RECC

**Figure 4:**
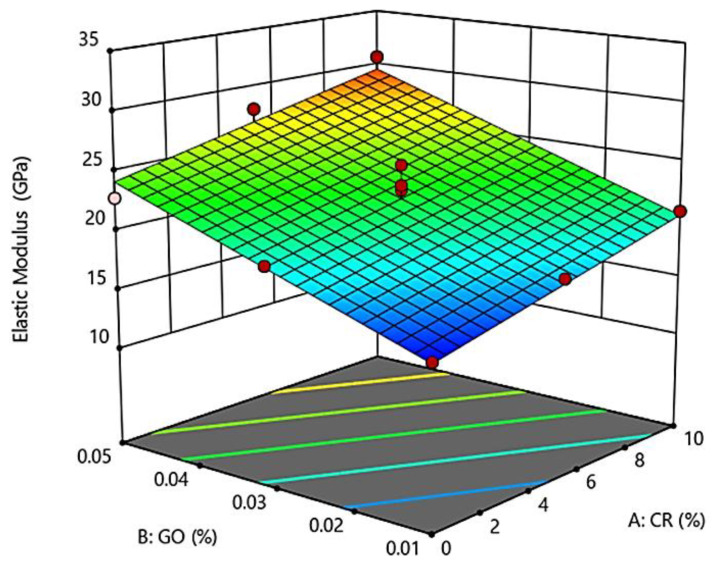
3D surface response for elastic Modulus of GO-RECC.

Figure 4 shows the 3D surface response representing the interaction effect of CR replacement and the addition of GO on the modulus of elasticity of the developed GO-RECC. The results show that the range of 0–5% CR replacement and 0.01–0.05% GO gave an elastic modulus range of 15.08 GPa to 30.7 GPa. As shown in the 3D surface response, with the concurrent increment of CR replacement and GO percentage, the elastic modulus of GO-RECC increased. It can also be observed that the addition of GO had more of an effect on the elastic modulus of GO-RECC compared to CR.

When the replacement content of CR increased, the elastic modulus increased. However, in contrast, the elastic and fiber nature of CR contributed to enabling GO-RECC to absorb more strain energy and increase ductile behavior [32]. However, the results in Figure 4 show that the elastic modulus increased with the increment of both CR and GO due to the amazing natural behavior of GO. It is known that GO has a natural elastic modulus value of around 32 GPa, representing outstanding performance among other cementitious materials [33]. Therefore, with the increment of GO in GO-RECC, the elastic modulus increased. The increase in elastic modulus can be caused by the GO decreasing the number of original shrinkage cracks. Furthermore, further studies showed that the presence of GO increases the area in the pre-peak state because of the increment in strain corresponding to peak stress. Nano-size cracks are propagated under load, and they form continuous micro cracks at the peak of the stress–strain curve, eventually leading to an increment in strain capacity [17].

### 3.4. Poisson’s Ratio of GO-RECC

**Figure 5:**
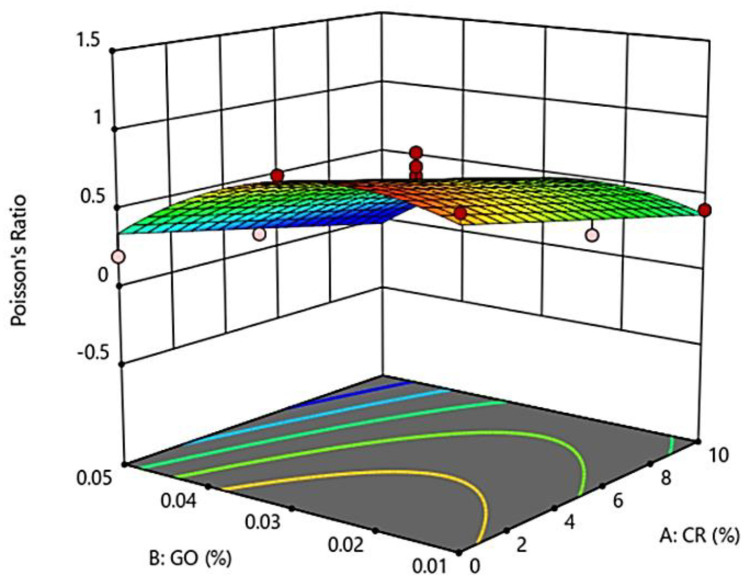
3D surface response for Poisson’s ratio of GO-RECC.

Results shown in Figure 5 illustrate the Poisson’s ratio of GO-RECC with the influence of a partial replacement of CR and the addition of GO. CR has a lower elastic modulus in nature compared to fine aggregates. When it comes to compressive stress, the lower deformation resistance causes it to experience larger axial compression. However, with the addition of GO, the strength improved. Figure 5 shows that, as the CR percentage increased, the Poisson’s ratio decreased gradually, while, with the increment of GO percentage, the Poisson’s ratio increased at first and then decreased after the content of GO exceeded 0.03%.

### 3.5. Drying Shrinkage of GO-RECC

**Figure 6:**
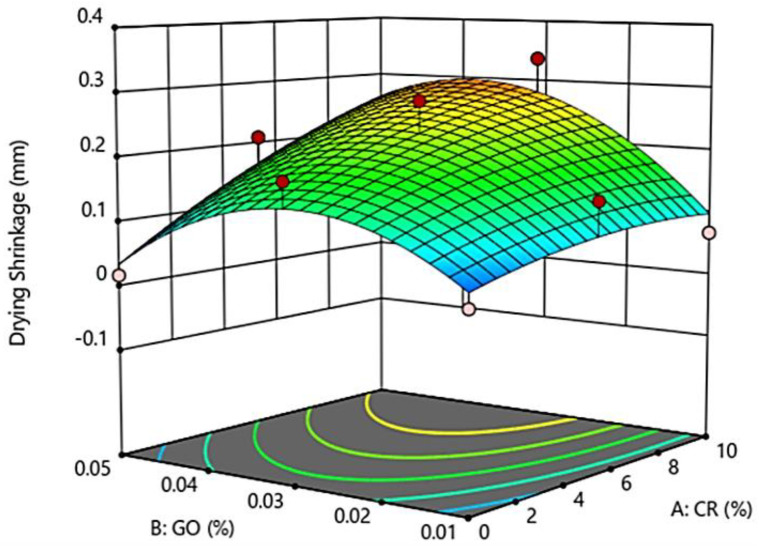
3D surface response for drying shrinkage of GO-RECC.

Figure 6 shows that the drying shrinkage of GO-RECC increased with an increase in the CR replacement; meanwhile, GO is shown to have an optimal effect on the drying shrinkage at around 0.03%, while the drying shrinkage decreased at percentages beyond 0.03%. 

The increase in drying shrinkage can be explained by the reduction in the amount of rigid river sand that could provide internal restraints to deformation due to drying shrinkage [34]. It is also reported that, with an increase in CR content, the porosity of the matrix also increases due to its hydrophobic nature, which eventually leads to higher drying shrinkage. 

On the other hand, the addition of GO generally decreased the drying shrinkage as the GO content went beyond 0.03%. It was found that GO in the ECC densified microstructure with nano-filler at a microscale helped reduce shrinkage and cracks, with energy absorption during the failure pattern at microscale [35]. It can be concluded that GO has a more significant influence on the drying shrinkage compared to CR.

### 3.6. Model Validation

Analysis of variance (ANOVA) quantifies the significance of a second-order polynomial function when a 5% significance level (*p* < 0.05) is achieved. From the analysis, it can be seen that the *p*-values of all RSM responses were below 0.05, indicating that the models were significant at the 95% confidence level (CI). This suggests that the models provide superior and accurate responses. 

Table 7 shows a summary of ANOVA for the developed models using RSM. It shows that the functions for compressive strength and elastic modulus were linear functions, while those for Poisson’s ratio and drying shrinkage were quadratic functions. The F-values for the compressive strength and elastic modulus models were 13.67 and 28.68, while the *p*-values for the same models were 0.0014 and 0.0001, respectively, i.e., less than 0.05. This clarifies the statistical significance of both models. Moreover, the *p*-value for each term in the model (CR and GO) was found to be less than 0.05, confirming the significant effect of CR and GO in both models (compressive strength and elastic modulus). As a result, A and B were both significant model terms at 95% CI, which were utilized to identify the effects of CR and GO on the compressive strength and elastic modulus of GO-RECC. The correlations between the factors A (CR) and B (GO) and their responses, in terms of compressive strength and elastic modulus, are given in the below-developed model equations, where Equation (1) represents compressive strength and Equation (2) represents elastic modulus.

Compressive Strength = 38.88 − 0.420 × CR + 131.92 × GO.(1)

Elastic Modulus = 12.66 + 0.541 × CR + 230.17 × GO.(2)

On the other hand, the functions for Poisson’s ratio and drying shrinkage were quadratic functions, as shown in Table 7. The models for Poisson’s ratio and drying shrinkage, as well as its terms A (CR) and B (GO), were significant with *p*-values less than 0.05. However, the terms B, AB, and A^2^ for the drying shrinkage model were insignificant with a *p*-value greater than 0.05. Therefore, the terms B, AB, and A^2^ did not have a significant effect on the drying shrinkage of the developed GO-RECC. The quadratic functions of Poisson’s ratio and drying shrinkage are shown in Equations (3) and (4), respectively.

Poisson’s Ratio = 0.482 − 0.0373 × CR + 33.405 × GO − 722.024 × GO^2^. (3)

Drying Shrinkage = −0.0729 + 0.0082 × CR + 14.797 × GO + 0.45 × CR × GO − 0.00079 × CR^2^ − 253.147 × GO^2^. (4)

Table 8 indicates the coefficient of determination for each RSM model. It is shown that the measured and predicted responses had a good correlation. *R^2^* values represent the goodness of fit for the models. This is the percentage of variance in the responses which the independent factors explain collectively. The *R^2^* values were 73%, 85%, 80%, and 80% of the variation for compressive strength, elastic modulus, Poisson’s ratio, and drying shrinkage respectively. The difference between adjusted *R^2^* and predicted *R^2^* for each model should be less than 0.2 [36], which indicates that the values of adjusted *R^2^* and predicted *R^2^* are in significant agreement. However, Table 8 shows that only compressive strength and elastic modulus had a difference value within 0.2, while Poisson’s ratio and drying shrinkage did not. 

The coefficient of variation (CV) is used to measure the variability of the experimental results to the overall mean. In addition, the adequate precision for all models exceeded 4, meaning that the design space defined by central composite design (CCD) could be navigated by the predicted models.

**Figure 7:**
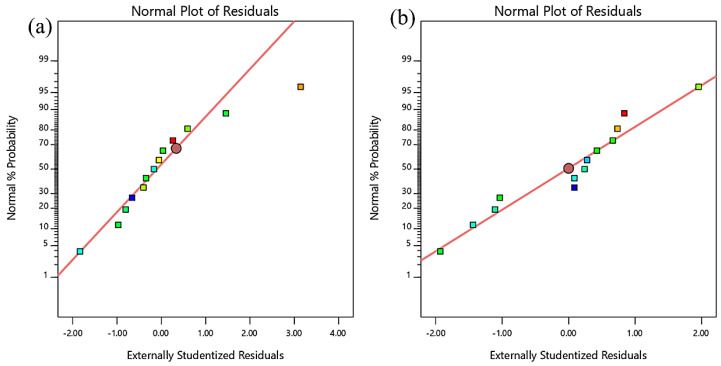
Normality plot of residuals for (**a**) compressive strength and (**b**) elastic modulus.

**Figure 8:**
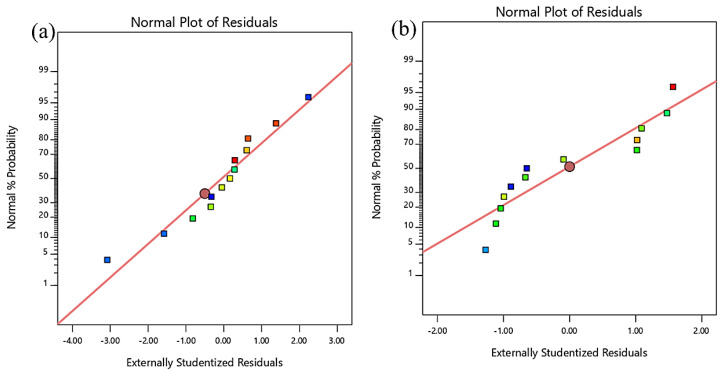
Normality plot of residuals for (**a**) Poisson’s ratio and (**b**) drying shrinkage.

The RSM models can also be validated through normality plots, as shown in Figure 7 and Figure 8. This is a graphical method used to evaluate the accuracy of the data. It is obvious that all the plots had points that fell close to the straight line, thus proving that the dataset was normally distributed, and the degree of randomness was the same for all fitted/predicted values. This proves the model’s fairness, as the residuals (the difference between experimental data and fitted values predicted by the model) were randomly distributed around zero. The entire normality plot shows that some responses differed from the predicted values. However, as long as these were within the red control limits, they are still acceptable.

For further clarification, it was reported that GO is the most lightweight, thinnest, strongest, stiffest, most flexible, most translucent, most waterproof, and most extendable material ever known [37,38]. Therefore, graphene motivated vast investigation interests due to its impressive characteristics, aiming at the discovery of new innovative nanomaterials in this construction era, with its application in ECC intriguing numerous scientists worldwide [39,40]. It is reported that the incorporation of rubberized ECC with GO revealed that the use of coarse crumb rubber is lower than the use of fine crumb rubber [41]. This indicates that the upsurge in the volume of crumb rubber marginally reduces the strength and penetrability in the concrete mass; thus, this developed mixture of rubberized ECC integrated with GO can be potentially used in the fabrication of several construction materials for applications such as road pavement/basement and filling voids, while it also enhances the freeze–thaw resistance and ductility [33]. 

## 4. Conclusions

The following conclusions can be drawn on the basis of the results obtained:

- The compressive strength of GO-RECC decreased when the CR replacement content increased. However, when GO was added concurrently, the strength increased as the natural behavior of GO caused the dispersion of all cement particles in void spaces, giving extra resistance of GO-RECC toward compression.
- The elastic modulus was tested with the increment with both CR and GO. It is concluded that GO played a vital role in increasing the elastic modulus of GO-RECC, whereby more strain energy was absorbed, and the ductile behavior was then increased.
- The Poisson’s ratio of GO-RECC increased at first and then decreased when the content of GO exceeded 0.03%. It is concluded that CR caused the developed ECC to experience a lower deformable resistance, leading to a larger axial compression; however, with the addition of GO, the strength was improved.
- The drying shrinkage of GO-RECC increased when the CR replacement content increased due to the increase in porosity induced by the hydrophilic nature of crumb rubber, while GO caused an increase in drying shrinkage at first, before decreasing after 0.03%.
- RSM enabled the targeted performance of GO-RECC to be achieved with reasonable precision across so many mixtures. The results showed that the quadratic models can predict the properties of new mixtures with reasonable accuracy by comparing the experimental results and the predicted statistical data.
- Cement paste and the surface of crumb rubber are hydrophilic and hydrophobic materials, respectively. Hence, the adhesion between cement paste and crumb rubber is weak, damaging the hardened characteristics of the crumb rubber matrix material and restricting the improvement and application of crumb rubber-based materials. Nevertheless, the surface characteristics of rubber also commonly experience a vital change from being toughly hydrophobic to hydrophilic, allowing adequate allocation in the cement matrix. It also has a lower mechanical strength than concrete, making crumb rubber a beneficial material, with numerous studies concentrating on adjusting crumb rubber to expand it as an additive of concrete. Therefore, an improvement in the performance and properties of crumb rubber in ECC incorporated with GO is highly imperative.

## Figures and Tables

**Table 1:** Chemical constituents of ordinary Portland cement (OPC) and fly ash (FA).

Constituents (%)	FA (%)	OPC (%)
SiO_2_	64.69	25.21
Al_2_O_3_	18.89	4.59
Fe_2_O_3_	4.9	2.99
CaO	5.98	62.85
MgO	1.99	1.7
Na_2_O	2.41	0.98
K_2_O	1.53	0.78
Loss on ignition	1.87	2.02
Specific gravity	2.3	3.15

**Table 2:** Properties of polyvinyl alcohol (PVA) fiber.

Type	Specific Gravity	Density (g/cm^3^)	Fiber Diameter (μm)	Fiber Length (mm)	Elastic Modulus (GPa)	Tensile Strength (MPa)	Aspect Ratio (l/d)
PVA	1.3	1.31	40	12	40	1600	462

**Table 3:** Physical properties of graphene oxide.

Form	Particle Size	Odor	Color	Concentration (wt.%)	Dispersibility	pH (4 mg/L Dispersion)
Slurry	6 μm to 33 μm	Odorless	Dark brown	2.5	Polar solvent	1.8 to 2.0

**Table 4:** Elemental analysis of graphene oxide.

Carbon	Nitrogen	Hydrogen	Oxygen	Sulfur
49–56%	0–1%	0–1%	41–50%	2–4%

**Table 5:** Mixture proportions with dosage of raw materials of GO-modified engineered cementitious composite (ECC). (CR—crumb rubber and SP—superplasticizer).

Mix	Cement kg/m^3^	FA kg/m^3^	Sand kg/m^3^	CR		Water kg/m^3^	SP kg/m^3^	PVA		GO	
				%	g			%	kg/m^3^	%	g
1	570	684	451.44	0	0.00	269.61	2.51	2.0	25.08	0.05	9.59
2	570	684	451.44	0	0.00	269.61	2.51	2.0	25.08	0.01	1.92
3	570	684	451.44	0	0.00	269.61	2.51	2.0	25.08	0.03	5.76
4	570	684	451.44	5	759.83	269.61	2.51	2.0	25.08	0.03	5.76
5	570	684	451.44	5	759.83	269.61	2.51	2.0	25.08	0.03	5.76
6	570	684	451.44	5	759.83	269.61	2.51	2.0	25.08	0.03	5.76
7	570	684	451.44	5	759.83	269.61	2.51	2.0	25.08	0.05	9.59
8	570	684	451.44	5	759.83	269.61	2.51	2.0	25.08	0.01	1.92
9	570	684	451.44	5	759.83	269.61	2.51	2.0	25.08	0.03	5.76
10	570	684	451.44	5	759.83	269.61	2.51	2.0	25.08	0.03	5.76
11	570	684	451.44	10	1519.67	269.61	2.51	2.0	25.08	0.05	9.59
12	570	684	451.44	10	1519.67	269.61	2.51	2.0	25.08	0.03	5.76
13	570	684	451.44	10	1519.67	269.61	2.51	2.0	25.08	0.01	1.92

**Table 6:** Mix combinations and response results.

Mix	Variables		Responses			
	CR (%)	GO (%)	Compressive Strength (MPa)	Elastic Modulus (GPa)	Poisson’s Ratio	Drying Shrinkage (mm)
1	0	0.05	45.81	22.8	0.2	0.017
2	0	0.01	38.2	15.08	0.81	0.027
3	0	0.03	42.26	19.7	0.87	0.183
4	5	0.03	40.8	20	0.6	0.197
5	5	0.03	44.21	23.4	0.64	0.227
6	5	0.03	39.5	25.13	0.82	0.177
7	5	0.05	43.3	28	0.12	0.21
8	5	0.01	40	18.1	0.46	0.147
9	5	0.03	41.67	20.45	0.67	0.18
10	5	0.03	40.19	23	0.73	0.283
11	10	0.05	40.1	30.7	0.16	0.237
12	10	0.03	38.4	22.4	0.2	0.337
13	10	0.01	35.18	20.7	0.4	0.067

**Table 7:** Mix ANOVA summary.

Responses	Source	Sum of Squares	Df	Mean Square	F-Value	*p*-Value	Significance
Compressive strength	Model	68.18	2	34.09	13.67	0.0014	Yes
	A (CR)	26.42	1	26.42	10.60	0.0086	Yes
	B (GO)	41.76	1	41.76	16.75	0.0022	Yes
	Residual	24.93	10	2.49			
	Lack of fit	11.61	6	1.93	0.5809	0.7372	No
Elastic modulus	Model	170.99	2	85.50	28.68	<0.0001	Yes
	A (CR)	43.85	1	43.85	14.71	0.0033	Yes
	B (GO)	127.14	1	127.14	42.65	<0.0001	Yes
	Residual	29.81	10	2.98			
	Lack of fit	11.44	6	1.91	0.4150	0.8391	No
Poisson’s ratio	Model	0.7146	3	0.2382	11.82	0.0018	Yes
	A (CR)	0.2091	1	0.2091	10.38	0.0105	Yes
	B (GO)	0.2360	1	0.2360	11.71	0.0076	Yes
	B^2^	0.2695	1	0.2695	13.37	0.0053	Yes
	Residual	0.1813	9	0.0201			
	Lack of fit	0.1519	5	0.0304	4.12	0.0974	No
Drying shrinkage	Model	0.0843	5	0.0169	5.54	0.0222	Yes
	A (CR)	0.0286	1	0.0286	9.39	0.0182	Yes
	B (GO)	0.0083	1	0.0083	2.72	0.1428	No
	AB	0.0081	1	0.0081	2.66	0.1467	No
	A^2^	0.0011	1	0.0011	0.3545	0.5703	No
	B^2^	0.0283	1	0.0283	9.31	0.0186	Yes
	Residual	0.003	7	0.0030			
	Lack of fit	0.0045	3	0.0045	2.34	0.2152	No

**Table 8:** Model term validation.

Response	Compressive Strength (MPa)	Elastic Modulus (GPa)	Poisson’s Ratio	Drying Shrinkage (mm)
SD	1.58	1.73	0.1419	0.0552
Mean	40.74	22.27	0.5138	0.1761
CV%	3.88	7.75	27.62	31.32
PRESS	42.08	45.19	0.4703	0.1047
−2 log likelihood	45.36	47.68	−18.65	−46.49
*R^2^*	0.7322	0.8515	0.7976	0.7983
Adjusted *R^2^*	0.6787	0.8218	0.7301	0.6542
Predicted *R^2^*	0.5481	0.7550	0.4751	0.0085
Adequate precision	12.4888	17.6181	10.9283	6.5946
BIC	53.05	55.38	−8.39	−31.10
AICc	54.03	56.35	−5.65	−20.49

Where CV: Coefficient of Variation, PRESS: Prediction Sum of Square, BIC: Bayesian information criterion, and AICc: The second order “corrected”(Akaike information criterion).
